# Investigating Microinvasive Intra‐Ocular Biopsy

**DOI:** 10.1111/ceo.14591

**Published:** 2025-08-04

**Authors:** Jared Ching, Shohei Kitahata, Hinako Ichikawa, Kazuaki Kadonosono

**Affiliations:** ^1^ Department of Ophthalmology and Microtechnology Yokohama City University Medical Centre Yokohama Japan; ^2^ Department of Engineering Science University of Oxford Oxford UK

**Keywords:** intra‐ocular biopsy, micro‐invasive biopsy, microneedles, retinoblastoma, uveal melanoma

## Abstract

**Background:**

Current minimally invasive methods of intraocular biopsy are confined to small gauge (G) needles and subretinal cannulae that can be prone to wound leakage at the biopsy site. We investigate the role of microneedles with internal diameters as small as 49G for intraocular biopsy in the posterior and anterior segments.

**Methods:**

Human uveal melanoma (UM 92–1) and retinoblastoma (Y79) cancer cell lines were aspirated using microneedles of different sizes with a vitrectomy set up, and cell viability was analysed. Suspensions of cancer cells with fluorescent microbeads were injected into the subretinal space of fresh ex vivo porcine eyes before simulating biopsy with microneedle retinal puncture, followed by imaging with optical coherence tomography (OCT) and histology. Anterior chamber puncture was performed with microneedles and imaged with anterior segment OCT and examined for aqueous leakage.

**Results:**

We find that microneedles can aspirate ocular cancer cells, both retinoblastoma and uveal melanoma, in vitro and retain a high level of cell viability, 72.83% (49G) compared to 97.00% (25G vitrector) in UM 92–1. Using an ex vivo porcine model, we find that a 49G microneedle creates a self‐sealing retinal wound that does not reflux microbeads of 200 nm in diameter. Further, we find that anterior chamber puncture with a microneedle via a corneal paracentesis results in no evidence of an aqueous leak (0%) compared to a leakage rate of 100% and 66% when using a 30G and 34G needle, respectively.

**Conclusion:**

A microinvasive approach to biopsy intraocular specimens is feasible, warranting further in vivo studies.

## 1 Introduction

Intraocular tumour biopsy is pursued when clinical evaluation is inconclusive or for the purposes of prognostication; however, there can be other indications such as patient preference or recurrence of an intraocular tumour [1]. Diagnostic uncertainty following clinical examination can occur in approximately 2.4%, warranting further investigation [2]. Biopsy techniques vary according to whether the lesion of interest is in the anterior or posterior segment [3]. Techniques to biopsy lesions in either segment generally utilise small gauge needles or vitrectomy cutter probes to take small samples of the lesion of interest for diagnostic purposes [1, 4, 5]. The least invasive technique is an aqueous tap, which can be used effectively in diagnosing iris ring melanoma and metastatic adenocarcinoma, for example, with 30 gauge (30G) needles [6, 7]. In the treatment of retinoblastoma seeds in the anterior chamber, it has been shown that 34G needles can be used to aspirate the contents of the anterior chamber and then the syringe swapped to deliver melphalan chemotherapy [8, 9].

Retinal microneedles, defined as having at least one dimension between 1 and 999 μm, have been developed to cannulate retinal vasculature with the goal of treating vaso‐occlusive diseases [10]. Patients treated with vitrectomy surgery and microneedle endovascular cannulation may impart clinical benefits [11, 12]. Such microneedles can be coupled with vitrectomy machines such as the Alcon Constellation Vision System and have an outer diameter of 50 μm and an estimated inner lumen diameter of 35 μm [10]. In comparison, the most commonly used and least invasive needles used for FNA include 27G, 413 μm and 30G, 312 μm outer diameters, respectively. Reports of a subretinal cannula (Polytip 25G/38G, MedOne, henceforth referred to as 38/41G polytip as per Table 1) used in retinal pigment epithelial (RPE) cell suspension and gene therapy delivery have an outer diameter of 38G (ca. 111 μm) [13, 14, 15]. The microneedle therefore has a 8.26, 6.24, and 2.22 times smaller outer diameter than the 27G, 30G and 38/41G polytip, respectively, which may result in smaller retinotomy and less invasive means to retrieve biopsy material.

**TABLE 1:** Summary of measurements made using light microscopy of needles, cannulae, and pipette tips used in the present study.

Needle	Bevel heel to bevel tip (i)	Bevel tip to point (ii)	Internal diameter	Outer diameter	Wall thickness	Estimated internal gauge	Nomenclature for present study
25G vitrectomy cutter	0.447	0.227	N/A	N/A	N/A	N/A	25G cutter
30 g needle	0.727	0.217	0.120	0.270	0.070	38G	30G needle
34G needle	0.477	0.083	—				34G needle
38G polytip	N/A	N/A	0.080	0.110	0.010	41G	38G/41G polytip
38G needle	0.120	0.020	0.060	0.110	0.040	43G	38G/43G needle
45G needle	0.110	0.039	0.030	0.050	0.010	49G	45G/49G needle
P200	N/A	N/A	0.500	0.950	0.19	25G	P200 tip

Although intraocular tumour biopsy techniques have progressed significantly over time with advances in microsurgical instrumentation, there have been no studies investigating the feasibility of microneedles. This study aims to understand the potential use of microneedles for intraocular biopsy and compare these to standard needles used in clinical practice.

## 2 Methods

### 2.1 Microscopy of Needles

Light microscopy of 25G cutter, 30G, 34G needles, the 38/41G polytip, 38G/43G needle, and 45G/49G needle were taken using a dissecting microscope Leica S9D with a Leica Flexacam C3 (Leica, Wetzler, Germany). The orthogonal needle profiles were imaged as well as the bore. Images were transferred to Image J for analysis. Scanning electron microscopy was undertaken with a Keyence VE‐8800 (Keyence Tokyo Laboratory, Tokyo, Japan) using carbon tape to secure the needles of interest. High magnification images were taken to examine the surfaces of the bevel of each needle.

### 2.2 Cell Culture

Human uveal melanoma 92–1 (UM 92–1) was cultured in RPMI 1640 medium supplemented with 10% fetal bovine serum, 2 mM L‐glutamine, 100 U/mL penicillin and 100 μg/mL streptomycin (Gibco, Massachusetts, USA). Cells were cultured at 37°C and 10% CO_2_ and proliferated in culture flasks (Nunc, Massachusetts, USA) as a monolayer to 80% confluency before subculture using trypsin to dissociate cells. Cells were cultured at 37°C and 10% CO_2_ and proliferated. Human retinoblastoma Y‐79 was cultured in RPMI 1640 medium supplemented with 10% fetal bovine serum, 2 mM L‐glutamine, 100 U/mL penicillin and 100 μg/mL streptomycin (KAC Co. Ltd., Nishigekkocho, Kyoto, Japan) in low adhesion culture flasks.

### 2.3 Surgical Instrumentation

For all experiments, the Constellation Vision System (Alcon, Fort Worth, TX, USA) was used. Pre‐made sterile 25 Gauge Vitrectomy packs were used with the Viscous Fluid Control Pack (BL7600, Alcon, Fort Worth, TX) that included a 10 mL syringe, syringe plunger, syringe cap, tubing set with syringe coupler, 20G, 23G, and 25G cannula. The needles used included a 30G, 34G, 25G/38G Polytip (MedOne, Saratosa, FL), Tochigi Seiko 0.11 mm prototype microneedle (Tochigi Seiko Co. Ltd., Tochigi, Japan), and the Tochigi Seiko 0.05 mm microneedle (Tochigi Seiko Co. Ltd., Tochigi, Japan). All experiments carried out in vitro utilised a HEPA filtered sterile hood alongside sterile surgical instruments.

### 2.4 Microneedle Wound Size

Under sterile conditions, 48 well cell culture plates were filled with 1.5 mL of UM 92–1 or RB Y79 cells at a density of 1 × 10^5^ per well. Each well filled with cell suspension was one empty well away from the adjacent well in all directions to avoid any cross contamination between wells. Polyvinyl chloride was placed over the 48 well cell culture plates until taut. Under video microscopy, each needle was used to puncture the polyvinyl chloride and microscopy images were taken for measurement of the wound size and determination of any cell suspension reflux.

### 2.5 Cancer Cell Viability Following Microneedle Aspiration

Cancer cell lines UM 92–1 and Rb Y79 were cultured in sterile conditions in adherent and non‐adherent flasks, respectively, and cultured until 80% confluent. Trypsin–EDTA was used to dissociate UM 92–1 before centrifugation at 1200 rpm for 5 min, and Y79 was centrifuged directly. The cells were resuspended in media and the corresponding cell density was calculated such that aspiration of 150uL of a cell suspension would contain 1 × 10^5^ cells. The Alcon Constellation Vision System was set up adjacent to the laminar flow hood, with sterile instruments placed within the hood. The sterile VFC packs were placed within the hood and set up so that each needle of interest could be used to extract cancer cell suspensions. After each 150 μL of cell suspension was aspirated, the needle was exchanged for a 20G cannula that is included in the VFC pack to inject the aspirated cell suspension into a corresponding well in the 96‐well plate. Fresh culture media was used to wash the VFC syringe between each repeat, using at least 2 mL to wash the inner walls and plunger of the syringe. Each needle aspiration was completed in triplicate, and the entire experiment was repeated three times at different time points and cell passage numbers.

Immediately after the cells were seeded in the 96 well plate, the middle well of each needle's suspension was assessed with the trypan blue exclusion assay. This was completed by removing 10 μL of cell suspension and mixing with 10 μL of trypan blue. Automated (TC‐20, Countess, Biorad) and manual counting with a haemocytometer (Watson Biolab, San Diegao, CA) of the total and viable cells was completed and the average taken. Following this, SYTOX Green nucleic acid stain 5 mM solution was used at a 1:1000 concentration to monitor cell death during cell culture on the same day as seeding, and after 1, 3, and 7 days of culture. Images were transferred to ImageJ (National Health Institutes, Bathesda, USA), processed in black and white and the same threshold applied to all images before quantification of fluorescent dots.

### 2.6 Immunostaining

Cells were washed in PBS before fixing in 4% paraformaldehyde (Polysciences, Pennsylvania, USA) for 15 min at room temperature and pressure. A further 3 × 5 min washes with PBS were performed before blocking (Blocking One, Nalacai Tesque, Kyoto, Japan) for 1 h at room temperature and pressure. The primary antibody‐fluorophore, phalloidin 594 (Thermo Fisher Scientific, Massachusetts, USA), was added at a concentration of 1:1000 with DAPI 1:100. The cells were washed with PBS 3 × 5 min once more before imaging.

### 2.7 Microscopy

Fluorescent microscopy was carried out with a Keyence BZ‐X710 Fluorescent Microscope (Keyence Tokyo Laboratory, Tokyo, Japan). Briefly, four channels including bright field, DAPI, GFP and TRITC were used to image all cell culture, retinal wholemount samples and retinal sections at magnifications of 4×, 10×, and 20×. All image processing was undertaken using the Keyence Analysis software package and Image J.

### 2.8 Ex Vivo Intraocular Biopsy Model

In order to study the retinal wounds created and possible cancer cell reflux from the subretinal space, we utilised an ex vivo pig eye model akin to Gatto et al. [16, 17] Pig eyes were harvested from freshly terminated animals 3–6 months old from local abattoirs. The eyes were delivered on ice with the extraocular muscles, conjunctiva, eyelids and tissues intact, and arrived at the laboratory within 2–4 h of termination. The pig eyes were mounted onto a polystyrene phantom head and washed with double distilled water (ddH_2_O). Using the Constellation Vision System (Alcon, Texas, USA), a 25 gauge three‐port vitrectomy was performed on each pig eye. A vitrectomy contact lens (Hoya, Tokyo, Japan) was used as the posterior viewing system. No vitrectomy was performed when injecting a high concentration (2 × 10^6^ cells in 100 μL culture media) of UM 92–1 cells into the subretinal space with a Tochigi Seiko 0.11 mm microneedle. The cannula was then exchanged for the needle of interest, 38/41G polytip, Tochigi Seiko 0.11 mm or Tochigi Seiko 0.05 mm needles, before intact retina distant to the initial puncture site and within the retinal bleb, before aspirating using the extract function. The viscous fluid control (Alcon, Texas, USA) set was used with the included 20 gauge cannula to aspirate cell suspensions into a 48 well cell culture plate after aspiration of the cell suspension. The extraction settings were set to a maximum of 200 mmHg for all needles except the Tochigi Seiko 0.05 mm, for which 65 mmHg vacuum was used.

### 2.9 Ex Vivo Anterior Chamber Tap Model

Pig eyes were used as described earlier, and a peripheral corneal puncture into the anterior chamber with 30G and 34G needles was undertaken. As the microneedles are too short to penetrate the entire corneal thickness, a partial paracentesis was fashioned with a 22.5° blade before the microneedle was carefully slid into the incision plane and then advanced into the anterior chamber. In all cases, 5% fluorescein sodium was used to assess for an aqueous leak with the Seidel Test. SD‐OCT was carried out in the regions of the corneal wound. For each wound, a fresh eye was used to avoid confounding by hypotony. Each puncture was repeated 6 times under surgical microscopy.

## 3 Results

### 3.1 Needle Characterisation

**FIGURE 1:**
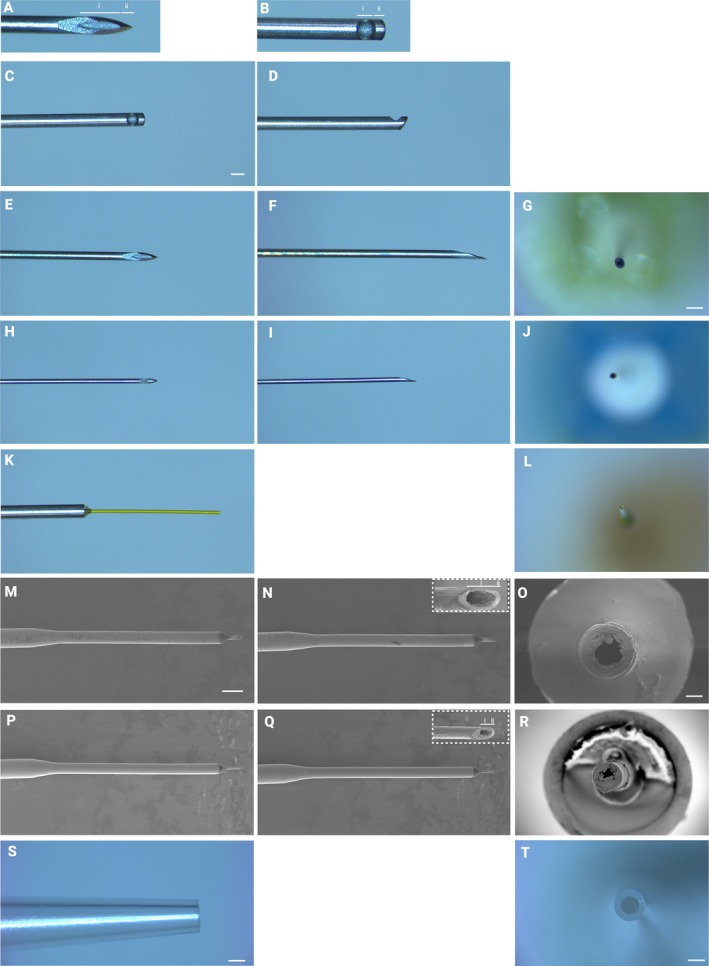
Multimodal imaging of needle tips used in the present study. (A) Photomicrographs of a 30G and (B) 25G vitrector probe, demonstrating distances defined by (i) bevel heel to bevel tip and (ii) bevel tip to point (this also applies to the inset for N, Q). A 25G vitrectomy probe showing different top down (C) and side (D) views of the cutter islet. The same views are demonstrated for the 30G (E, F), 34G (H, I) and 25G/38G MedOne Polytip (K). Electron microscopy images showing top down and side views of the 38G/43G (M, N) and 45G/49G (P, Q) microneedles. Side view of a standard laboratory tip for a 200 μL (P200) pipette (S) used as control. Cross sectional photomicrographs for the 30G, 34G, 38G/41G needles and control pipette are shown in (G, J, L, T), respectively. Electron microscopy of the cross section of the microneedles following laser cutting for the 38G/43G (O) and 45G/49G (R), note the burrs formed within the lumen from this process. The scale bar in (C) represent 500 μm and also apply to images (D–F, H, I, K). The scale bar in (G) represent 500 μm and apply to (J, L). The scale bar in M represent 500 μm and also apply to images (N, P, Q). The scale bar in (O) represent 28.5 μm and apply to (R).

A range of needles currently used in clinical practice were measured using light microscopy and electron microscopy in order to obtain estimates of the point and bevel dimensions and cross‐sectional dimensions. The results of this are summarised in Figure 1 and Table 1. Additional measurements were taken for the 38G/41G and 45G/49G microneedles, for which the length that would be usable for the purposes of penetrating the tissue of interest was 0.430 and 0.310 mm, respectively. Given that manufacturers of microneedles use in‐house mathematical algorithms to estimate the gauge of the manufactured needles rather than direct measurements, it is clinically useful to have estimated reference values for surgical planning. The control conditions were a standard laboratory pipette tip used with a 200 μL variable fluid pipette.

The most relevant dimensions for performing subretinal biopsies of lesions such as suspected uveal melanoma are the total distance between the point of the needle and heel of the bevel. Although a vitrectomy cutter does not share the structural features of a hypodermic needle, it has an islet, similar to a needle bore, for which measurements have been applied in a similar fashion (Figure 1). As a vitrectomy cutter or needle enters the retina, the minimum depth to expose the tissue of interest is found by adding the bevel heel to bevel tip distance and the bevel to point distance. Therefore, a 25G vitrectomy cutter must be inserted at a distance of 674 μm below the retina to fully expose its islet to biopsy a lesion. Needles currently used in clinical practice would require a depth of 944, 560, 140, and 149 μm for the 30G, 34G, 38 g/43G and 45G/49G needles, respectively. The 38G/41G polytip cannula has a flat tip and therefore does not need to traverse a specific distance within the tissue to maximise tissue exposure to the lumen of the cannula.

### 3.2 Microneedle Puncture Model

**FIGURE 2:**
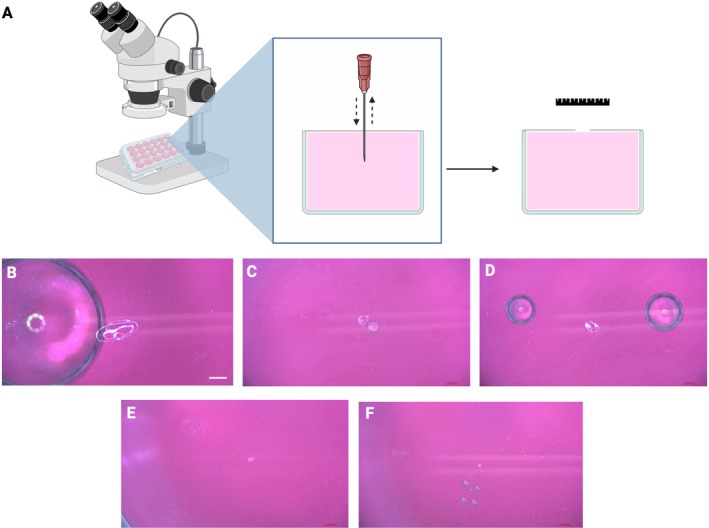
Schematic and representative images of polyvinylchoride (PVC) puncture. (A) Schematic showing 48 well plate filled with uveal melanoma cell line 92–1 with polyvinylchloride covering the top of the plates. The needle of interest punctures the centre of the PVC sheet and is withdrawn, before measurement of the defect formed. Representative images of the puncture created by (B) 25G cutter, (C) 30G needle, (D) 38G/42G Polytip, (E) 38G/43G needle, (F) 45G/49G needle. Note the air bubbles that are created by needle puncture in (B–D) due the size of the needle. Pre‐existing bubbles present during the 49 well plate preparation in (F).

Polyvinyl chloride (PVC) was wrapped over a 48 well plate with uveal melanoma 92–1 suspensions filled to the brim of each well, making each well airtight. Each needle was used to puncture a full thickness hole through PVC and then imaged to take measurements (Figure 2). These measurements are summarised in Table 2 as vertical and horizontal measurements. It was found that larger needles of interest caused consistent air bubbles below the level of the PVC sheet.

**TABLE 2:** Summary of mean vertical and horizontal estimates of wounds created by different needles through a polyvinylchloride sheets.

	Vertical (mm)		Horizontal (mm)	
Needle	Mean	SD	Mean	SD
25G cutter (on)	0.56	0.06	1.17	0.52
30G needle	0.37	0.04	0.84	0.50
38G/42G Polytip	0.24	0.02	0.29	0.09
38G/43G needle	0.12	0.03	0.18	0.03
45G/49G needle	0.20	0.10	0.12	0.05

### 3.3 Cell Viability Following Microneedle Aspiration

**FIGURE 3:**
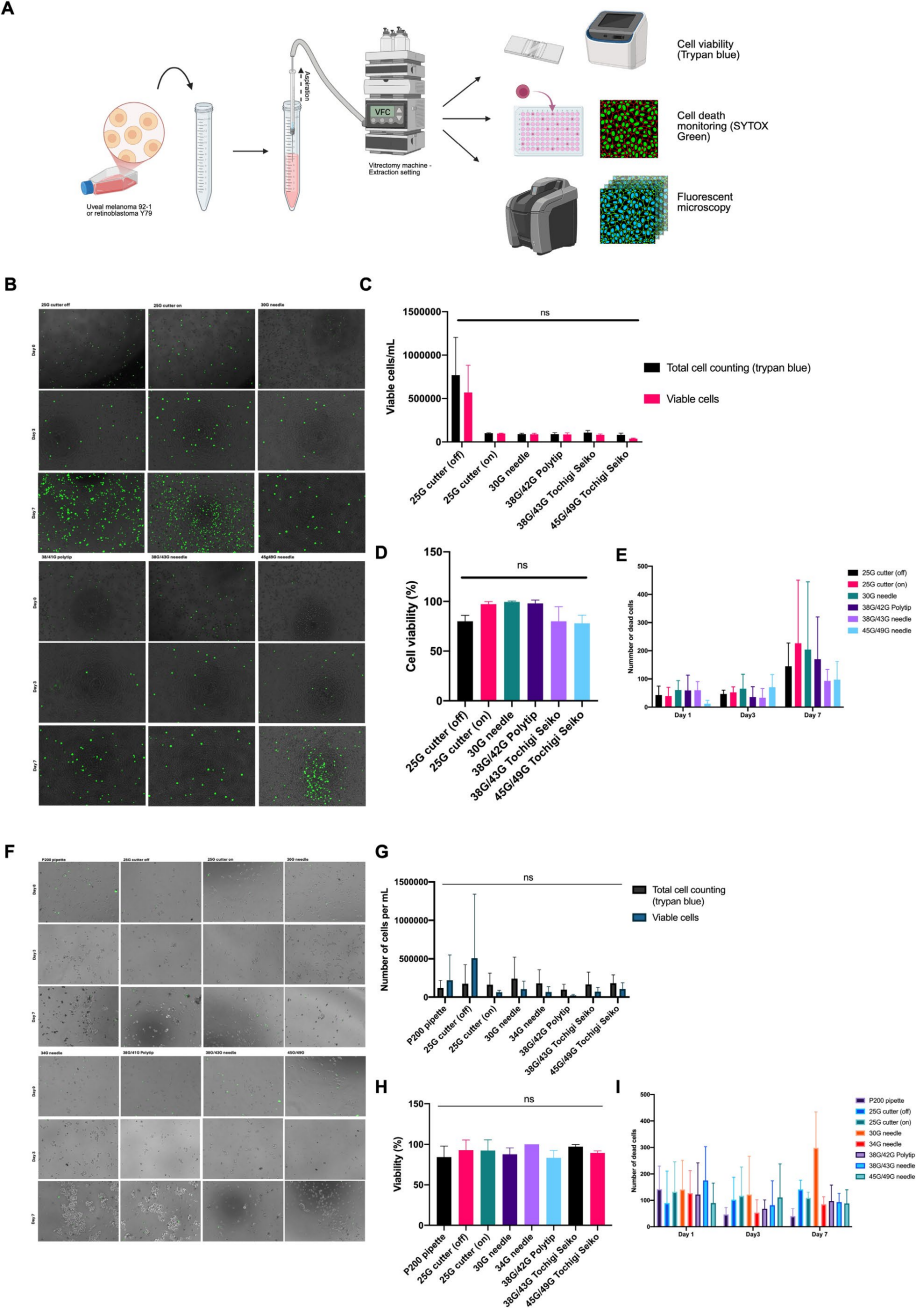
Aspiration of cancer cell suspensions through microneedles. (A) Schematic demonstrating research protocol. Briefly, cells are dissociated in the case of uveal melanoma 92–1 and aspirated through the needles of interest using a viscous fluid control system from the Constellation Vision System. Cells are counted using an automated counter (Countess, Biorad) and haemocytometer. SYTOX green is used to monitor cell death and cells are fixed with paraformaldehyde for immunocytochemistry. Photomicrographs of merged bright field and GFP merged channels, showing (B) 92–1 cells and (F) Y79 cells cultured over 7 days following aspiration with the needles of interest. Dead cells appear green. There was no significant difference in cell viability and total cell counts found (C, D). (E) SYTOX green dead cell counting revealed a significant increase in dead cell staining of 92–1 cells at day 7 (*p* < 0.0001 2 way ANOVA with Tukeys post hoc test). Similarly for retinoblastoma Y79, there was no significant difference in (G) total cell counts found and (H) cell viability. (I) SYTOX green dead cell counting revealed a no significant difference between days 1, 3 and 7.

As a surrogate for examining the effectiveness of undertaking a microneedle biopsy of a suspicious retinal or subretinal lesion, we examined cell viability following aspiration of a high density cancer cell suspensions using the trypan blue exclusion assay and SYTOX green dead‐cell nuclear stain. When aspirating uveal melanoma cell line 92–1 through the 25G vitrectomy cutter on and off, the 30G needle, 38G/41G polytip, 38G/43G needle, and 45/49G needle, the immediate mean cell viability measured with trypan blue was 83.00% (standard deviation ±9.54%), 97.00% (±2.65%), 98.33% (±2.89%), 94.00% (±10.39%), 83.30% (±16.71%), and 72.83% (±15.23%), respectively (Figure 3D). The mean total number of cells detected for each condition was 6.12 × 10^5^ (±8.47 × 10^5^), 1.07 × 10^5^ (±2.22 × 10^4^), 9.61 × 10^4^ (±2.80 × 10^4^), 8.92 × 10^4^ (±3.26 × 10^4^), 1.01 × 10^5^ (±4.44 × 10^4^), and 9.83 × 10^4^ (±5.61 × 10^4^), respectively (Figure 3C). The mean number of live cells detected was 4.59 × 10^5^ (±6.07 × 10^5^), 1.03 × 10^5^ (±1.95 × 10^4^), 9.41 × 10^4^ (±2.55 × 10^4^), 8.21 × 10^4^ (±3.38 × 10^4^), 7.97 × 10^4^ (±1.68 × 10^4^), and 4.49 × 10^4^ (±3.29 × 10^4^), respectively.

The same experiments were carried out for the retinoblastoma cell line Y79 with the addition of a 200 μL pipette as a further true control and 34G needle given its current clinical use in retinoblastoma anterior chamber seed management [9]. When aspirating retinoblastoma cell line Y79 through the 200 μL pipette, 25G vitrectomy cutter on and off, the 30G needle, 34G needle, 38G/41G polytip, 38G/43G needle, and 45/49G needle, the immediate mean cell viability measured with trypan blue was 84.33% (±13.65%), 92.66% (±12.70), 92.33% (±13.27%), 87.66% (±8.08%), 100% (±0.00%), 83.33% (±9.07%), 97.33% (±2.51%), 89.33% (±2.51%), respectively (Figure 3H). The mean total number of cells detected for each condition was 1.21 × 10^5^ (±9.76 × 10^4^), 1.74 × 10^5^ (±2.50 × 10^5^), 1.62 × 10^5^ (±1.50 × 10^5^), 2.42 × 10^5^ (±2.79 × 10^5^), 1.79 × 10^5^ (±1.78 × 10^5^), 9.93 × 10^4^ (±6.92 × 10^4^), 1.65 × 10^5^ (±1.61 × 10^5^), and 1.81 × 10^5^ (±1.09 × 10^5^), respectively (Figure 3G). The mean number of live cells detected was 2.21 × 10^5^ (±3.28 × 10^5^), 5.08 × 10^5^ (±8.33 × 10^5^), 6.56 × 10^4^ (±2.34 × 10^4^), 1.04 × 10^5^ (±1.03 × 10^5^), 6.82 × 10^5^ (±6.83 × 10^5^), 2.49 × 10^4^ (±1.18 × 10^4^), 7.30 × 10^4^ (±5.46 × 10^4^), and 1.07 × 10^5^ (±8.15 × 10^5^), respectively.

In order to gain insights into delayed cell following aspiration of the cell suspensions, SYTOX green dead cell assay was used to monitor the cells in culture over 7 days. Notably, no significant difference in dead cell numbers was found at day 1 and 3 for 92–1 cells and days 1, 3 and 7 for Y79 cells (Figure 3E). However, at day 7 for 92–1, there was a statistically significant increase in dead cells detected (*p* < 0.0001 2 way ANOVA with Tukeys post hoc test) (Figure 3I). The detailed results of this are presented in Supporting Information S1 and S2.

### 3.4 Cytoskeletal Changes Following Microneedle Biopsy

**FIGURE 4:**
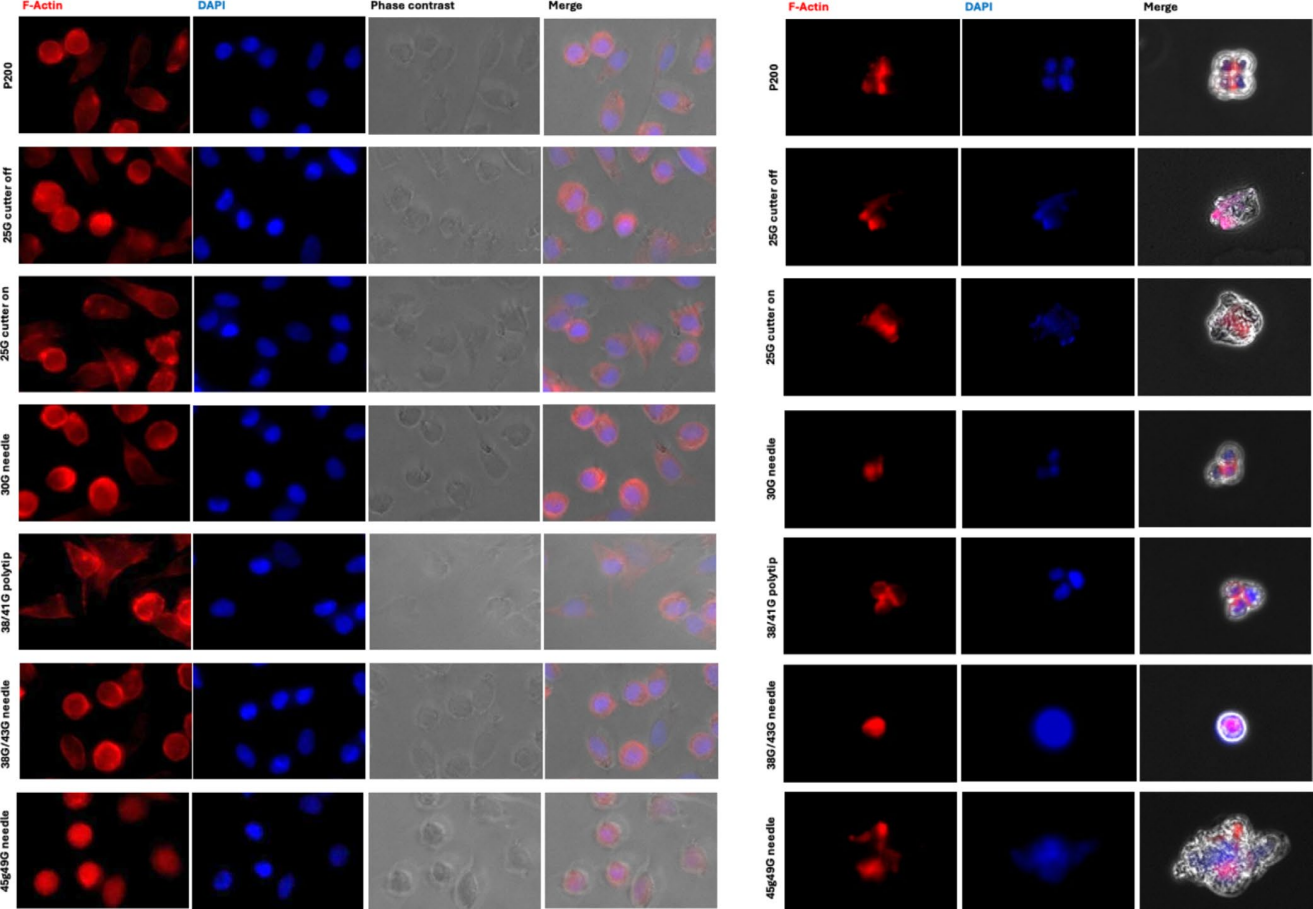
Immunocytochemistry for uveal melanoma 92–1 (adherent cell culture) and retinoblastoma Y79 (non‐adherent cluster culture) after 24 h incubation following extraction as a cell suspension. Left panel demonstrates 92–1 cells and the right Y79.

As it is known that cells can undergo shear stress during passage through a small lumen, we examined a common cytoskeletal protein to screen for any obvious changes in structural organisation that can occur in conditions of high shear stress. After aspiration of the cell suspension using each condition, the cells were cultured for 24 h prior to fixation. The 92–1 cell line demonstrated no changes in the ability of cells to adhere and proliferate on phase contrast imaging. The typical pleiomorphic cell phenotype and large nucleoli did not appear to vary compared to the control conditions or in any of the conditions (Figure 4). Further, the distribution of F‐actin expression did not appear to differ from the control conditions or between conditions. Similarly, Y79 cells did not demonstrate any apparent derangement in F‐actin arrangement.

### 3.5 Ex Vivo Retinal Puncture Model

**FIGURE 5:**
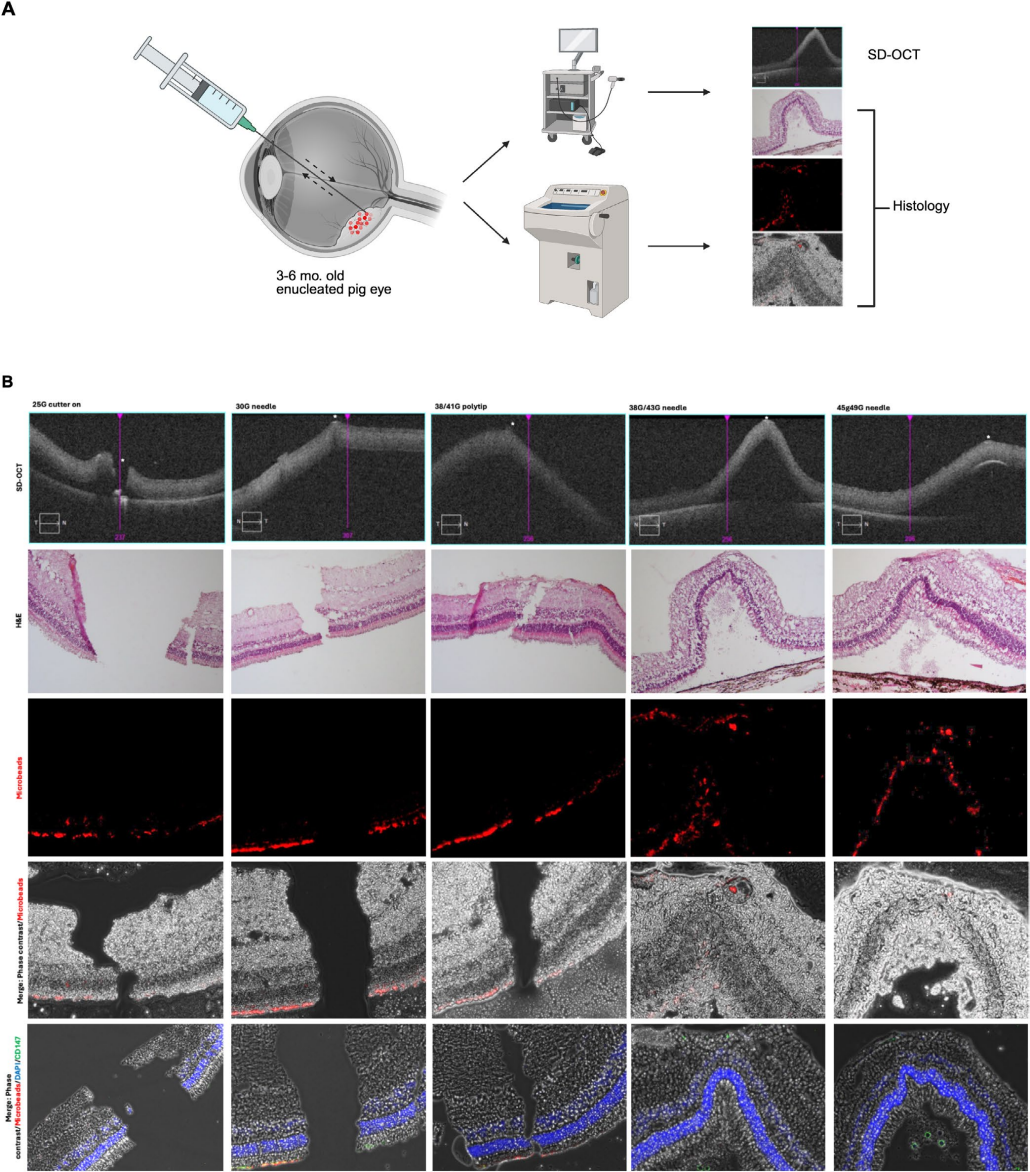
Ex vivo analysis of retinal puncture to simulate transretinal biopsy. (A) Schematic showing ex vivo retinal puncture model; briefly, fluorescent microbeads are injected into the subretinal space before needles of interest are used to puncture the retina and then withdrawn from the eye. The eyes are subsequently fixed with paraformaldehyde before SD‐OCT imaging and cryosectioning. (B) Multimodal imaging of the puncture sites marked with diathermy intraoperatively. OCT images show that the retinal wound created by the 25G vitrector is clearly visible; however, this is not mimicked with the other needles. Haematoxylin and eosin staining reveals the extent of the retinal wound present with the microneedles appearing to form self‐sealing wounds. Fluorescent microbeads appear to migrate to the pre‐retinal space after retinal puncture with the 38G/43G needle but not the smaller 45G/49G. The larger needles did not have any microbeads associated with the edges of the retinal wound nor the pre‐retina; however, this has been thought to possibly be due to the washing out effect of the infusion and tissue preparation process. Antibody staining with CD147 was largely negative, with no evidence of tumour cell engraftment. Further, in merged images of fluorescent microbeads and CD147, the serial tissue sections show that the beads are washed away during processing, impairing analysis in this setting.

In order to characterise the retinal wound created by using different biopsy needles or cannula, we developed an ex vivo puncture model. A suspension of fluorescent microbeads and tumour cells is injected into the subretinal space using a 38G/43G without performing a vitrectomy, followed by marking the site of simulated biopsy with diathermy. The needle or cannula is then used to puncture the marked retina before the eye is fixed for histological analysis. All conditions were repeated three times. In all instances, a small plume of suspension containing microbeads was visualised after withdrawal of all needles/cannula except the 45G/49G needle. Immediately after the procedure, SD‐OCT images were taken and the retinal wounds were imaged using the diathermy marks on the inner retina as guidance (Figure 5). SD‐OCT showed clear retinal wound when the 25G cutter was used to puncture the retina. It was found that without turning on the 25G vitrectomy cutter, the angled blunt tip required significant force to create a retinal wound and therefore the cutter was switched to create the wound. Aspiration of the subretinal bleb of fluorescent microbeads and cancer cell suspension was inevitably aspirated leading to a flat appearance on SD‐OCT (Figure 5). For all other needles/cannula, the retinal wound was not clearly visible on OCT (Figure 5). We hypothesise that although the surgical wounds were clearly visible intraoperatively, following surgery, the globes inevitably lose intraocular pressure despite the valved trochars being left in situ and the retinal wounds may be masked by retinal relaxation secondary. Following surgery, the ex vivo pig eyes were fixed in paraformaldehyde and cryoprotected with sucrose solutions before being dissected as shown in Figure 5.

Haematoxylin and eosin (H&E) serial section demonstrated full thickness wounds were created by the 25G cutter, 30G needle and 38G/43G needles. Due to variability of cryosectioning, it was found that the edges of the wound created by the 30G needle tended to fold, giving a false impression of the size and extent of the retinal wound. In contrast, the microneedles 38G/43G and 45G/49G did not create a visible wound on histology and it was found that a small subretinal bleb was required in order to more accurately define the wound histologically. In all serial sections of 10 μm slices, a clear wound could not be identified; however, fluorescent microscopy of the unprocessed tissue slices revealed that the fluorescent microbeads remained attached on the outer retina in all conditions. Notably, it was found that the microbeads formed a tract along the retinal puncture site that led to epiretinal microbeads being deposited when using the 38G/43G but not the 45G/49G needle. Staining for the human cell marker CD147 and DAPI revealed that the microbeads were washed away during processing and very few 92–1 cells could be detected, possibly due to a lack of integration into the retinal tissues.

### 3.6 Ex Vivo Corneal Puncture Model

**FIGURE 6:**
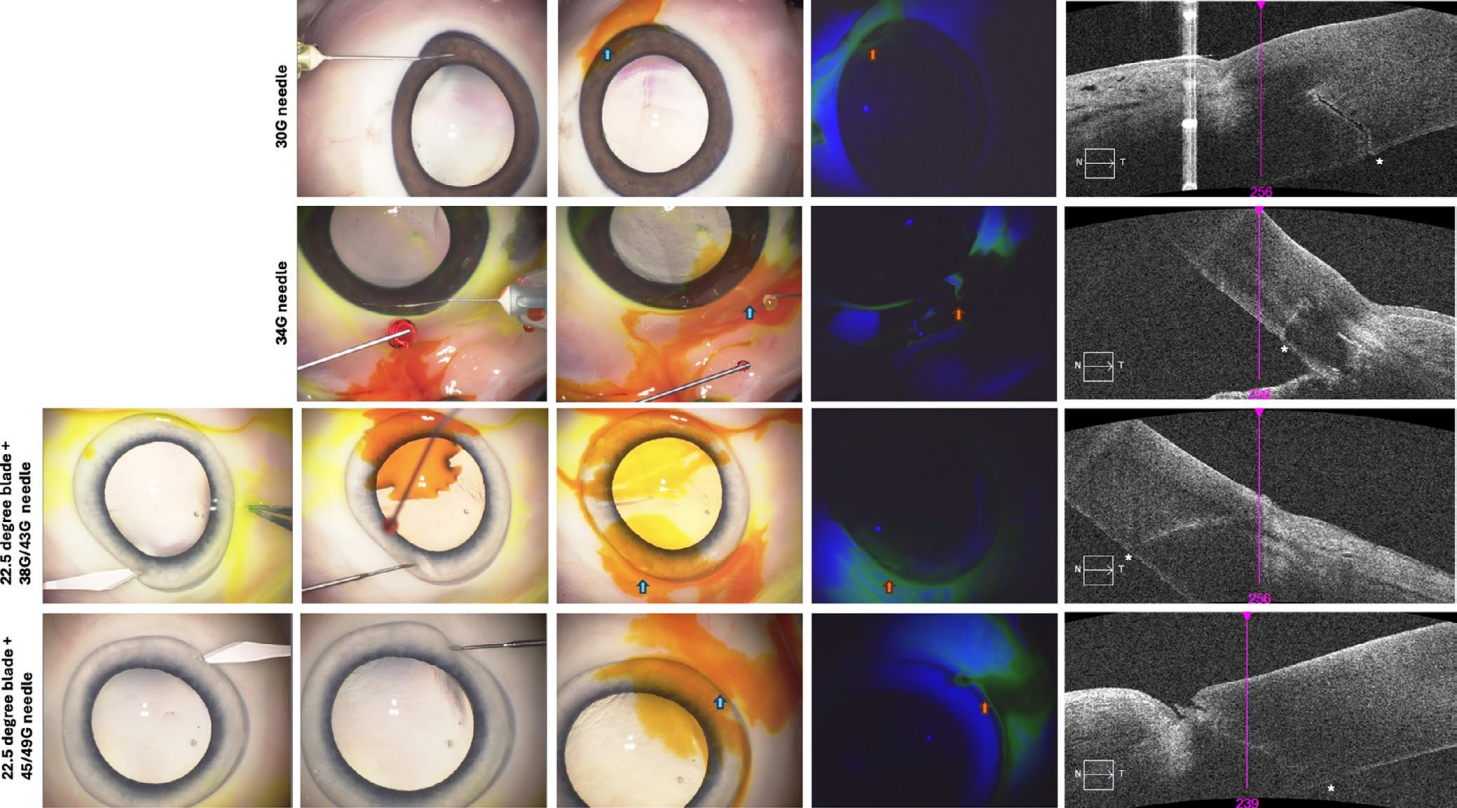
Ex vivo anterior chamber puncture model. Direct anterior chamber puncture was performed using 30G and 34G needles, finding that Seidel test was positive (fluorseceine is diluted at the site of puncture, creating a light yellow streak within the fluorosceine area). Anterior segment SD‐OCT confirms that wound architecture and that the wounds formed by 30G and 34G have a small degree of wound gape on the inner cornea. In contrast, the microneedles 38G/43G and 45G/49G needles do not form any aqueous leak after anterior chamber puncture. Due to the length of the microneedles, a partial paracentesis is formed (as shown with the 22.5° blade) before the anterior chamber is punctured. SD‐OCT confirms that the corneal wound is just visible but appears to be full apposed.

In order to assess the risk of extraocular spread following an anterior chamber paracentesis, we developed an ex vivo model to assess self‐sealing of different needles. We used fluorescein and cobalt blue light to perform a Seidel Test for aqueous leakage. We repeated each puncture 6 times, finding that the 30G needle causes leakage in 6 of 6 paracenteses (100%) and the 34G needle causes leakage in 4 of 6 (66%) (Figure 6). Representative videos of this are provided in Supporting Information S3–S6. In contrast, when using a combination of a 22.5^o^ blade and the 38G/43G or 45G/49G, no aqueous leakages were detected, 0 of 6 paracenteses (0%) in both instances. Anterior segment SD‐OCT was carried out at the site of the needle wounds. Notably, the commonly used 30G needle created full‐thickness wounds with a variable amount of wound gape and irregular tract. The 34G needle, in comparison, created a more regular, straighter wound with almost no wound gape visible. In contrast, the 38G/43G or 45G/49G created very small inner corneal wounds where there appeared to be a minimal corneal tract and no evidence of wound gape.

In order to further evaluate the effect of the paracentesis with a 22.5° blade, we performed a partial paracentesis before puncturing the anterior chamber with the 30G and 34G. In all instances of the 22.5° blade and 30G, there was aqueous leakage which stopped after several seconds in a variable fashion. However, with the 22.5° blade and 34G needle, minimal aqueous leakage was detected in all 6 cases. This indicates that surgical technique may play an important role in wound construction; for example, the technique describes forming a long intracorneal tract to avoid any extraocular aqueous following the intracameral withdrawal and injection procedures [9].

## 4 Discussion

Herein we present early data demonstrating that a microinvasive approach is feasible for intraocular biopsies of suspicious lesions using in vitro and ex vivo methods. Strengths of this study include the use of equipment that is widely used in the clinical setting. Further, we employ human cancer cell lines to study the effect of such cells passing through microneedles. Whilst the data we present is promising, we acknowledge that it is limited by the lack of in vivo data and the use of solid tumour samples.

Histologically, uveal melanoma and retinoblastoma are solid tumours that can develop cystic spaces owing to necrosis or develop calcification, respectively. Therefore, in vivo, these tumours may not behave like a single cell suspension, as we have investigated in the present study. This may result in paucicellular or heterogeneous samples when employing a microinvasive approach and possibly depend on tumour characteristics such as chronicity, thickness, and mutations such as BAP1 loss. Primary tumour cells may also be more sensitive to shear forces when passing through a microscopic needle bore, which may result in inadvertent derangement in protein expression, for example. Therefore, in clinical practice, we predict that there may be a learning curve and optimisation phase in order to implement such a technique. The target volume or size of sample will also vary according to the goal of the biopsy, such as for diagnostic or prognostic purposes.

Currently, the smallest needles used in posterior tumour biopsy are 30G for manual fine needle aspiration and 34G needles for anterior chamber retinoblastoma manual aspiration and melphalan delivery. Using a vitrectomy approach, the 38G/41G polytip has been used to effectively biopsy primary vitreoretinal lymphoma in the subretinal space [18] and uveal melanoma without vitrecomy [19]. The biopsy site of the 38/41G needle is thought to be self‐healing, with OCT images from Petrovic et al. confirming this with fundus and OCT images [18]. The present data suggest that a 38/41G cannula creates a full thickness retinal defect that possibly undergoes fibrosis to seal rather than being truly self‐sealing (Figure 5). Although our histological analysis with fluorescent microbeads and UM 92–1 cells does not demonstrate cellular reflux, we demonstrate a minimally visible biopsy site with a very fine needle tract.

The effectiveness of vitrector assisted biopsy has been evaluated in vitro by Ulltang et al., finding that using a 23G versus 25G did not significantly affect the number of hepatocytes retrieved; however, cellular and tissue fragment yields were high with higher cut rates up to 6000 cpm [20]. In comparison, Jiang et al. found that when using 20G cutters, lymphoma cells lost cell viability with cut rates above 600 cpm [21]. Given these findings, we used a maximal cut rate of 6500 cpm and compared this to aspiration alone. Interestingly, although there was no significant difference in the yield and viability of cells retrieved using the cutter on, without any cutting, the 25G vitrector aspirated a more inconsistent number of cells with more variable viability, particularly when aspirating suspensions of uveal melanoma 92–1. Interestingly, when comparing the viability and cell counts of hypodermic needles and microneedles used in this study, there was no significant difference with the vitrector or control 200 μL pipette. Since 92–1 cells grow adherently as a monolayer and Y79 as a non‐adherent cluster suspension, our results indicate that it may be possible to use microneedles to aspirate anterior chamber seeds of retinoblastoma, as described by Munier et al. [8].

In order to gain insight into shear stress that may adversely affect biopsied cells, we examined the expression of F‐actin. Cucina et al. previously showed that vascular endothelial cells undergo cytoskeletal re‐modelling when undergoing laminar flow where F‐actin exhibits a diffuse pattern, losing its regular distribution [22]. In our experiments, we found that there were no adverse changes to F‐actin distribution in both 92–1 and Y79 cells from any of the microneedles tested.

The emergence of cell free DNA (cfDNA) in the diagnosis of retinoblastoma has permitted minimally invasive diagnosis via an anterior chamber tap of aqueous humour [23]. Berry et al., in their seminal work, removed 100 μL of aqueous using a 32G needle for histopathological analysis and next generation sequencing, finding the presence of tumour‐derived cfDNA. In our study, we utilised a smaller 34G needle as described by Munier et al. for tumour aspiration and injection of melphalan [9], finding that the microneedles can retrieve a comparable percentage of viable retinoblastoma cells in confluent cluster suspension. Our ex vivo model of anterior chamber tap shows that both 30G and 34G needles can be associated with a significant risk of aqueous leakage compared to microneedles using the Seidel Test in freshly enucleated porcine eyes. The wound architecture is also visibly different when comparing the microneedles to the 30G and 34G wounds, indicating that wound gape is more likely in the standard hypodermic needles. Our results indicate that retrieving cfDNA from the anterior chamber with a microinvasive approach may be feasible.

## 5 Conclusions

A microinvasive approach to surgical biopsy of intraocular fluids and lesions is feasible, warranting further in vivo studies in animal models. We demonstrate the potential for microinvasive biopsy to form self‐healing wounds that may not cause any extratumoural spread and thus enhance the safety profile of such procedures. This study utilises clinically validated equipment and instrumentation, making any future translation into the clinical setting efficient. Our findings suggest that such micro biopsy techniques may have clinical uses in other medical fields beyond ocular oncology.

## Conflicts of Interest

K.K. holds a patent for the 45G/49G needle described in this work (Patent No. 5777074).

## Supporting information


**Data S1:** Supporting Information.

**Video S1:** Supporting Information.

**Video S2:** Supporting Information.

**Video S3:** Supporting Information.

**Video S4:** Supporting Information.

## Data Availability

The data that supports the findings of this study are available in the Supporting Information of this article.
