# Supplementary material for: Investigating Microinvasive Intra‐Ocular Biopsy
Source: Clin Exp Ophthalmol. 2025 Aug 4;53(8):996–1007. doi: 10.1111/ceo.14591 (PMC12596421; doi:10.1111/ceo.14591)
Supplement: Supplementary file 1 — Data S1: Supporting Information. [file CEO-53-996-s004.docx]

**Supplementary information**

**S1**


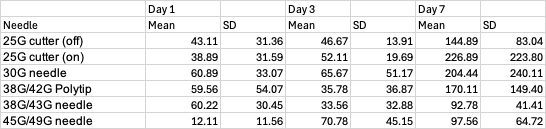


Dead cell counting using SYTOX green for 92-1 cells cultured over 7 days.

**S2**


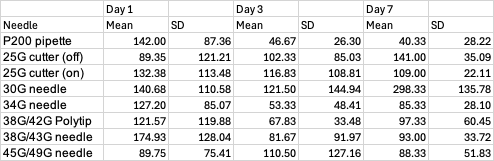


Dead cell counting using SYTOX green for Y97 cells cultured over 7 days.

**S3**

Anterior chamber paracentesis with a 30G needle followed by Siedel’s test using Fluorosceine.

**S4**

Anterior chamber paracentesis with a 34G needle followed by Siedel’s test using Fluorosceine.

**S5**

Partial paracentesis with a 22.5^o^ blade followed by a 38G/41G anterior chamber puncture and Siedel’s test using Fluorosceine.

**S6**

Partial paracentesis with a 22.5^o^ blade followed by a 45G/49G anterior chamber puncture and Siedel’s test using Fluorosceine.
